# An unexpected allergic reaction with *Saccharomyces boulardii*: a case report

**DOI:** 10.1186/2045-7022-4-S3-P100

**Published:** 2014-07-18

**Authors:** Ozgur Kartal, Fevzi Demirel, Abdullah Baysan, Mustafa Gulec, Sait Yesillik, Metin Uyanýk, Ugur Musabak, Osman Sener

**Affiliations:** 1 Gulhane Military Medical Academy and Medical School, Division of Immunology and Allergic Diseases, Turkey; 2 Gulhane Military Medical Academy and Medical School, Department of Clinical Chemistry, Turkey

## Introduction

*Saccharomyces boulardii* (*S.boulardii*), known as a nonpathogenic yeast probiotic shows it's efficacy in inflammatory and infectious diseases of the gastrointestinal tract safely. This report presents an allergic reaction and positive skin test in a patient who takes *S.boulardii* as an antidiarrheal therapy.

## Case Report

A sixty-year old male patient was admitted to the hospital with itchy rash on both ankles within 1.5 hours after ingesting *S.boulardii* 250 mg capsule and nifuroxazide 100 mg capsule. Hyperemic macular lesions surrounding the ankles were seen on physical examination. Oral antihistamine, oral and topical steroid therapies were initiated. Skin tests with suspected drugs were performed a month later. The prick and intradermal tests were performed with *S.boulardii* and nifuroxazide. The intradermal test with *S.boulardii* was detected positive at 1/10 W/vol concentration. However, skin tests were found to be negative with nifuroxazide.

## Conclusion

Although *S.boulardii* is known a safe drug in the treatment of some gastrointestinal disorders, it can not be referred completely reliable on the basis of allergic reactions. Previously, only one allergic reaction was reported effecting gastrointesinal system. Our case is important in clinical practice, because the first skin manifestation of *S.boulardii* allergy was presented and confirmed by skin test.

